# Dysregulation of Small Nucleolar RNAs in B-Cell Malignancies

**DOI:** 10.3390/biomedicines10061229

**Published:** 2022-05-24

**Authors:** Martijn W. C. Verbeek, Stefan J. Erkeland, Vincent H. J. van der Velden

**Affiliations:** Department of Immunology, Erasmus MC, University Medical Center Rotterdam, Doctor Molewaterplein 40, 3015 GD Rotterdam, The Netherlands; m.w.c.verbeek@erasmusmc.nl

**Keywords:** B-cells, acute lymphoblastic leukemia, chronic lymphocytic leukemia, B-cell non-Hodgkin’s lymphoma, multiple myeloma, small non-coding RNA, small nucleolar RNA, snoRNA

## Abstract

Small nucleolar RNAs (snoRNAs) are responsible for post-transcriptional modification of ribosomal RNAs, transfer RNAs and small nuclear RNAs, and thereby have important regulatory functions in mRNA splicing and protein translation. Several studies have shown that snoRNAs are dysregulated in human cancer and may play a role in cancer initiation and progression. In this review, we focus on the role of snoRNAs in normal and malignant B-cell development. SnoRNA activity appears to be essential for normal B-cell differentiation and dysregulated expression of sno-RNAs is determined in B-cell acute lymphoblastic leukemia, chronic lymphocytic leukemia, B-cell non-Hodgkin’s lymphoma, and plasma cell neoplasms. SnoRNA expression is associated with cytogenetic/molecular subgroups and clinical outcome in patients with B-cell malignancies. Translocations involving snoRNAs have been described as well. Here, we discuss the different aspects of snoRNAs in B-cell malignancies and report on their role in oncogenic transformation, which may be useful for the development of novel diagnostic biomarkers or therapeutic targets.

## 1. Introduction

B-cells are essential for the humoral immune response [1]. B-cells mature via strictly-regulated mechanisms in the bone marrow (BM) from stem cells to naive B-cells (antigen-independent) and mainly in lymph nodes from naive B-cells to memory B-cells and plasma cells (antigen-dependent) [1]. Each B-cell maturation stage can undergo an oncogenic transformation, resulting in a variety of B-cell malignancies, ranging from acute lymphoblastic leukemia (ALL), chronic lymphocytic leukemia (CLL), B-non-Hodgkin’s lymphoma (B-NHL) to multiple myeloma (MM). B-cell malignancies present with a wide range of molecular abnormalities and recurrent cytogenetic aberrations [2,3,4,5], often related to the B-cell specific processes, such as VDJ gene arrangements, somatic hypermutation and class switching.

Small noncoding RNA (sncRNA) are highly abundant RNAs that have key regulatory functions in various cellular processes [6]. MicroRNAs (miRNAs), the most studied endogenous sncRNAs, are small single-stranded RNAs of approximately 22 nucleotides (nts) in length and target messenger RNAs (mRNAs) for post-transcriptional silencing [7]. Another class of sncRNAs are the small nucleolar RNAs (snoRNAs), which are 50–300 nts in length and are mainly localized in the nucleoli. SnoRNAs are responsible for post-transcriptional modification of ribosomal RNAs (rRNAs), transfer RNAs (tRNAs), and small nuclear RNAs (snRNAs) [8].

Several studies have shown that snoRNAs are involved in oncogenesis [9,10,11,12,13]. For instance, a large study including over 10,000 samples across 31 cancer types identified 46 snoRNAs that showed clinical relevance in at least 12 cancer types. SnoRNAs were associated with DNA damage response and mitotic nuclear division, suggesting a role in cancer initiation and progression. Furthermore, genes that are associated with the biogenesis and functions of snoRNAs were dysregulated in multiple types of cancer [11]. Other studies, involving colorectal carcinoma and pancreatic cancer, showed that snoRNAs were dysregulated and were involved in disease progression [12,13]. In this review, we discuss the role of snoRNAs in normal B-cell differentiation as well as their dysregulation in various B-cell malignancies.

## 2. SnoRNAs

SnoRNAs are part of a group of uridylic-acid-rich small RNAs that are primarily localized in the nucleus. In the past decades, an increasing number of snoRNAs and related functions have been discovered. To date, more than 700 different human snoRNAs are identified and listed in databases including the snoRNA-LBME, snOPY and the SnoDB database [14,15,16]. Based on structure, function and/or subcellular localization, snoRNAs can be divided in three main groups: C/D box snoRNAs, H/ACA box snoRNAs, and small Cajal bodies-specific RNAs (scaRNAs) [14,17,18,19,20].

## 3. Structure and Biogenesis of C/D Box snoRNAs

**Figure 1:**
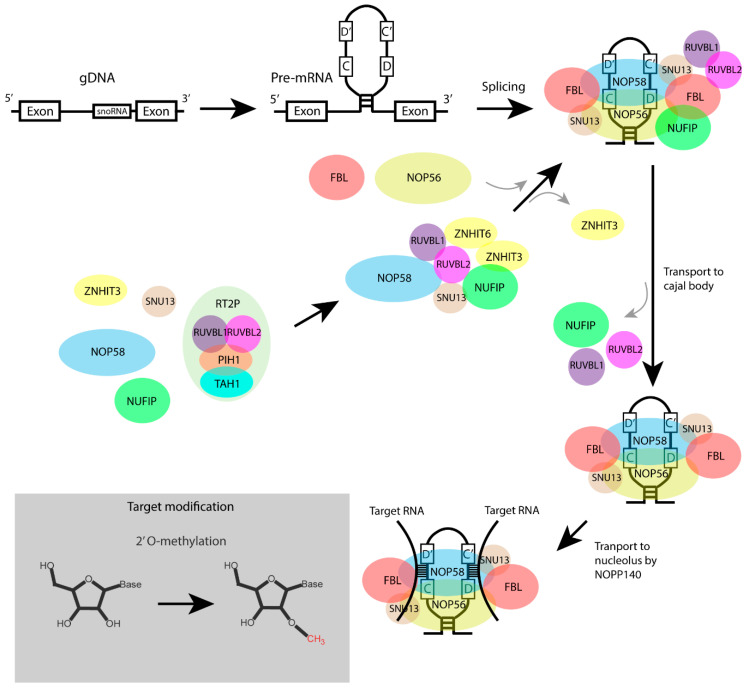
Biogenesis of C/D box snoRNAs. C/D box snoRNAs are located near the 3′ splice site of the intron. During transcription, C/D box snoRNAs form their characteristic secondary RNA structure. The formation of C/D box snoRNPs requires different assembly factors, including the RUVBL1-RUVBL2-TAH1-PIH1 (R2TP) complex [31,40]. The RUVBL1 and RUVBL2 subunits of the R2TP complex bind, together with nuclear fragile X mental-retardation-interacting protein 1 (NUFIP), zinc finger HIT domain-containing protein 3 (ZNHIT3), and ZNHIT6, the core protein nucleolar protein 58 (NOP58) and small nuclear ribonucleoprotein 13 (SNU13) to form a new protein complex [35]. This newly assembled complex then binds and recruits fibrilarin (FBL), which replaces ZNHIT3 in the complex. The complex binds the C/D box snoRNA sequence during the splicing of the pre-mRNA. RUVBL1, RUVBL2, NUFIP, and ZNHIT6 are released from the complex and the immature snoRNP is translocated to the Cajal body for further maturation before it is translocated to the nucleolus. The mature C/D box snoRNAs are associated with four proteins, SNU13, NOP56, NOP58, and FBL [41,42]. C/D box snoRNA guide the 2′O-methylation of targets rRNAs.

The C/D box snoRNAs are the largest group of snoRNAs, with more than 400 entities present in the human genome [14]. C/D box snoRNAs are 50–100 nts in length and they contain the highly-conserved motifs box C (RUGAUGA) and box D (CUGA) [21,22]. C/D box motifs are critical for the characteristic secondary helix–bulge–helix structure, the so-called K-turn [21,23]. In addition to the C/D box, these snoRNAs contain less conserved copies of the C and D motifs, the C’ and D’ motifs [24]. In between the C, C’ motifs and D, D’ motifs are the so-called guide sequences that direct the snoRNA to reverse complementary motifs within the target rRNAs [17,23] (Figure 1).

C/D box snoRNA are mainly located in introns of host genes coding for proteins of the ribosomal biogenesis, proteins involved in translation, or proteins with a nuclear or nucleolar function [25]. The biogenesis of C/D box snoRNAs largely depends on the splicing of pre-mRNA [26,27]. Within the intron, C/D box snoRNAs are preferentially positioned approximately 70 nts of the 3′ splice site [28]. The transcription of C/D box sno-RNAs is performed by RNA polymerase II and includes the addition of a 5′ monomethyl guanosine CAP structure [29]. After transcription, the snoRNA sequence in the pre-mRNA is bound to a complex of various proteins [30,31,32,33], and the small nucleolar ribonucleoprotein (snoRNP) complex then recruits nucleolar protein 56 (NOP56) and fibrillarin (FBL). Subsequently, the pre-mRNA is spliced and the immature snoRNP complex is then transported to the Cajal bodies, where the 5′ cap is hypermethylated by the trimethylguanosine synthase (TGS)–coilin complex [34]. Next, the mature snoRNPs are transported to the nucleoli by the 140 kDa nucleolar phosphoprotein (NOPP140) [35,36]. The snoRNA guides the snoRNP complex to the target rRNA by the interaction with reverse complementary motifs [24,37,38,39], whereas the methylation reaction is performed by the methyltransferase FBL [24] (Figure 1).

## 4. Structure and Biogenesis of H/ACA Box snoRNAs

**Figure 2:**
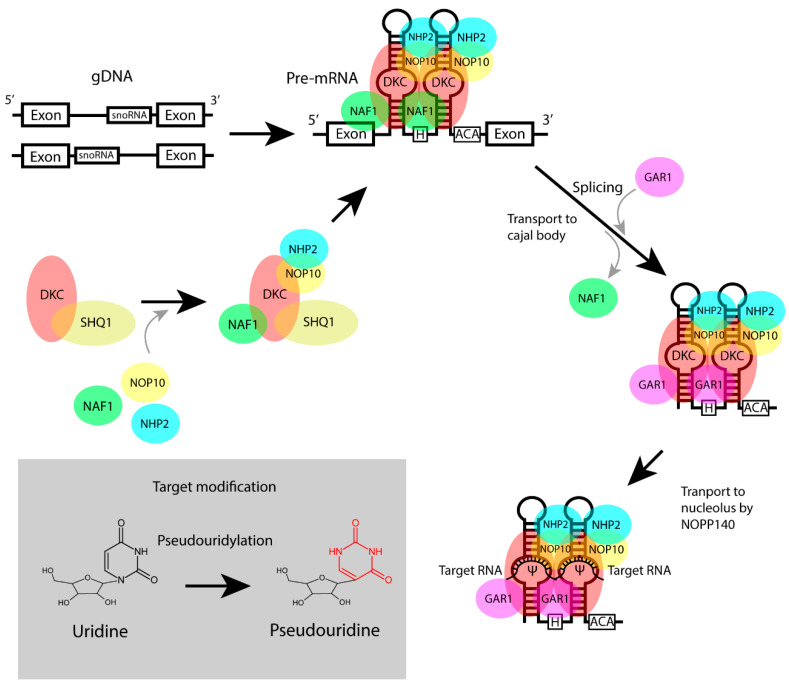
Biogenesis of H/ACA box snoRNP. The generation of a H/ACA box snoRNP complex starts with the binding of the SHQ1 protein to dyskerin (DKC1). Subsequently, three additional proteins (nuclear assembly factor 1 (NAF1), H/ACA RNP complex subunit 3/nucleolar protein 10 (NOP10) and H/ACA ribonucleoprotein complex subunit 2 (NHP2)) bind the DKC1-SHQ1 complex, mediated by the RUVBL1-RUVBL2-TAH1-PIH1 (R2TP) complex [20,35]. After the release of SHQ1, this new complex is then transported to the nucleus where it binds the snoRNA sequence on the pre-mRNA. After splicing, the immature H/ACA box snoRNP is translocated to the Cajal bodies where NAF1 is exchanged by H/ACA ribonucleoprotein complex subunit 1 (GAR1). The H/ACA box snoRNP is translocated to the nucleolus by NOPP140, where it can perform the pseudouridylation of target rRNA. Figure modified from reference [35].

H/ACA box snoRNAs are the second main group of snoRNAs. Up to now, more than 200 different human H/ACA box snoRNAs have been discovered [14], all consisting of a specific H-(ANANNA) and an ACA-box motif [18,23,37]. The ACA-box motif is always located three nucleotides from the 3’ end of the snoRNA strand [18]. The H/ACA box snoRNAs possess a characteristic secondary structure, consisting of two 60–75 nucleotide-long hairpins [18,23,36,37], in which the H-box motif is located in-between [18,23,37]. Both hairpins contain a pseudouridylation pocket which is essential for target interaction, as it contains the reverse complementary sequence to the target rRNA [18,23,36] (Figure 2).

H/ACA box snoRNA can be positioned both near the 5′ and the 3′ splice site of the intronic region [28]. After transcription of the host gene and splicing of the pre-mRNA, the immature H/ACA box snoRNA is translocated to the Cajal bodies, where it binds to a protein complex, resulting in H/ACA box snoRNP [35]. The H/ACA box snoRNP is translocated to the nucleolus, where it pseudouridylates target RNA. The H/ACA box sno-RNAs guide the RNP complex to the target rRNAs, whereas the pseudouridylation reaction is carried out by the pseudouridine synthase dyskerin (DKC1) [23,24,37,39].

## 5. Structure and Biogenesis of scaRNAs

ScaRNAs are the third type of snoRNAs and are, other than C/D box and H/ACA box snoRNAs, specifically localized in the Cajal bodies. ScaRNAs include C/D box or H/ACA box domains [21,23,43], but there are also compound scaRNAs that contain both C/D boxes and H/ACA boxes [38,39]. Currently, 24 human scaRNAs are known, four C/D box scaRNAs, four C/D-H/ACA mixed scaRNAs, and 16 H/ACA box scaRNAs [19]. The Cajal bodies localization of scaRNAs is due to two specific Cajal body localization signals: the CAB Box domains [21,23,43,44]. Each CAB box domain contains a CAB consensus (UGAG) sequence in the hairpin structures of the H/ACA or C/D domain [43]. ScaRNAs are associated with COILIN, a Cajal body’s specific protein, and H/ACA or C/D box core proteins [45,46]. C/D box and H/ACA box scaRNAs are assembled as their C/D box and H/ACA box snoRNA counterparts [19], whereas the assembly of composite box scaRNAs is less well understood. ScaRNPs are involved in the 2′O-methylation (C/D box scaRNA) and/or pseudouridylation (H/ACA box scaRNA) of snRNAs, which are involved in splicing of pre-mRNAs [17].

## 6. Canonical Functions of snoRNAs

The 2′-O methylation and pseudouridylation of rRNA and snRNAs are an important part of the maturation of spliceosomal RNPs [47], and these modifications increase their stability [48,49]. Modifications on target RNAs are not randomly distributed, but are found on highly conserved nucleotides [50]. Besides the protection against degradation, modifications on rRNA regulate the binding of ribosomal proteins to the rRNA to form the ribosomes [51,52,53]. Ribosomes were thought to be homogenous structures in the cell [52], but it was recently shown that ribosomes occur in different compositions. These differences occur by the high heterogeneity of post-transcriptional modifications of rRNA [48,50,52,54,55,56,57,58] and subsequent binding of different ribosomal protein analogs to the ribosomes, a concept known as ribosomal heterogeneity [52]. These different ribosomes have a unique regulation of translation. For example, different ribosomes have specific preferences for the translation of specific mRNAs [52,53]. Furthermore, it has been shown that ribosomes can be partially modified and that there are different modification signatures in cellular stress conditions compared to normal [56] and between health and disease state [55,57,59]. These findings suggest an important snoRNA-driven mechanism of translational control and protein expression [48].

## 7. Noncanonical Functions of snoRNAs

Besides the canonical housekeeping and regulatory functions, snoRNAs may have other functions as well. First, it was discovered that some snoRNAs can be processed by DICER and that the sno-derived small RNAs (sdRNA) have miRNA-like functions [60,61,62]. sdRNAs processed from C/D snoRNAs are mainly at 17–19 nts or larger than 27 nts in size and predominantly originate from the 5′ end of the snoRNA [62,63]. In contrast, sdRNAs processed from H/ACA snoRNAs are predominantly 20–24 nts in length and originate mainly from the 3′ end of the snoRNA [62].

Second, it has been shown that there is a direct interaction between argonaute (AGO) 1 and AGO2 with NOP56 and FBL in HEK293 cells, indicating a possible link between RNA-induced silencing complex (RISC) and snoRNA function [64]. In 2008, Ender et al. identified that the human snoRNA scaRNA15/ACA45 is processed by DICER into a small regulatory RNA, which is bound to AGO1/AGO2 and silences the expression of cyclin-dependent kinase 19 (CDK19, CDC2L6) [60]. By regulating the translation of CDK19, scaRNA15/ACA45 plays a potential role in cell proliferation. Thus, some sdRNAs are loaded in RISC and have important regulatory functions. Consequently, dysregulation of snoRNA may not only affect splicing and ribosomal function but may also directly have an effect on cellular proliferation.

## 8. Noncanonical Targets of snoRNAs

In recent years, new targets of snoRNAs have been discovered [65]. First, like rRNAs, tRNAs undergo post-transcriptional modifications [65,66]. It was found that the human snoRNAs SNORD97 and scaRNA97 have a sequence complementarity with the anticodon loop of tRNA^Met^ at cytidine 34 (C34) [65]. This may indicate that C34 is a target for 2′O-methylation by SNORD97 and scaRNA97. Indeed, it was shown in vertebrates that C34 is methylated, and that the modification prevents the cleavage of tRNA^Met^ by angiogenin. A single and double SNORD97/scaRNA97 knockout human HAP-1 cell model showed that the methylation of C34 is guided by SNORD97 in cooperation with scaRNA97. However, this double knockout cell model did not inhibit proliferation of HAP-1 cells, suggesting that C34 methylation is not essential for canonical tRNA^Met^ function.

Second, Huang et al. found that SNORD50A negatively regulated the mammalian mRNA 3′ processing complex by binding to the polyadenylation site and competing with Pre-mRNA 3’-end-processing factor FIP1 (FIP1) [67]. The mRNA 3′ processing complex is involved in the maturation of mRNA and the regulation of translation [68,69]. Indeed, downregulation of SNORD50A optimized the processing of mRNA 3′-ends, which increased mRNA levels [65,69]. These data suggest that these snoRNAs may have a regulatory function on the mRNA level as well [67].

## 9. SnoRNAs in Normal Hematopoiesis

Mounting evidence shows that snoRNAs are essential for normal hematopoiesis [10,70]. Genes associated with snoRNA functions and biogenesis were found mutated in hematopoietic disease. For instance, *DKC1* is mutated in inherited syndromes including X-linked dyskeratosis congenita (X-DC) and Hoyeraal–Hreidarsson syndrome, a clinically severe variant of dyskeratosis congenita (DC) and characterized by severe bone marrow failure [71,72]. Strikingly, only the expression of SNORNA15 and SNORNA67 was downregulated in DKC1-mutant CD34+ cells compared with normal CD34+ cells [70]. Variable expression of different H/ACA snoRNAs was found in X-DC patient cells with different mutations and the dysregulation of H/ACA box snoRNAs in X-DC patients was cell type dependent. These results indicate the complexity of snoRNA dysregulation in the X-DC patients [70]. In addition, it has been shown that CD34+ cells with a catalytically inactive mutant of DKC1 (DKC1^D125A^) were greatly impaired in their capacity to differentiate in myeloid and erythroid colony assays as compared to CD34+ control cells [70]. An acquired mutation at arginine 525 of DKC1 (DKC1^R525H^) and loss of function mutations in the DDX41 gene, a factor that is required for proper snoRNA processing in hematopoetic stem and precursor cells (HSPCs), contribute to hematopoietic defects leading to myelodysplasia [73]. DDX41 mutated cells showed decreased expression levels of snoRNAs, resulting in reduced pseudouridylation of rRNA target uridines and impaired formation of ribosomes [73]. R525H is hypomorphic for DDX41 function and may allow for HSC survival, whereas the rapid proliferative hematopoietic progenitors are more sensitive to loss of DDX41 activity due to their dependency on high protein synthesis [73]. Loss of snoRNA function in HSPCs causes cell cycle arrest and apoptosis caused by dysregulation of pathways including p53 stress response, phosphatidylinositol 3-kinase (PI3K/AKT and Wnt/β-catenin [73]. Together, these results indicate that snoRNA activity is essential for normal hematopoiesis.

Only a few studies describe the expression levels of snoRNA in normal hematopoiesis. In the study of Warner et al., the expression of snoRNA was analyzed in different hematopoietic cell fractions and hierarchical clustering analysis of snoRNA expression data showed grouping of the samples dependent on cellular lineage and developmental state [10]. For instance, it was found that two imprinted snoRNA-containing loci, namely the DLK-DIO3 locus (containing a large number of sncRNAs including 41 snoRNAs) and the SNURF/SNRPN locus (containing 82 snoRNAs), were expressed at high levels in CD34+ progenitor cells and strongly downregulated during granulocytic differentiation. Interestingly, the DLK-DIO3 locus snoRNAs were also expressed at reduced levels in B and T-cells, whereas the expression of SNURF/SNRPN snoRNAs remained high [10], suggesting for cell type-specific functions of these snoRNAs. The regulation of snoRNA expression was not correlated with splicing of host genes. In addition, no evident correlation between snoRNA expression and the expression of the host gene has been found in hematopoietic cells [10]. These results indicate that other, yet unknown factors are involved in the regulation snoRNA expression and stability. In another study, snoRNA expression was compared between different types of normal mature B-cells [74]. Germinal center (GC), naïve (N), marginal zone (MZ) and memory B-cells (SM), all isolated from tonsils, expressed similar levels of snoRNA, whereas peripheral blood B-cells (pBC) showed a distinct snoRNA expression pattern with reduced expression of SNORD116-1, SNORD116-23, SNORD116-29, SNORD94, and SNORA36A [74]. Liuksiala et al. showed that SNORA25 (ACA25) and SNORA61 (ACA61) are highly expressed in naïve B-cells compared to HSCs, naive T cells and pediatric leukemia (T-ALL, BCP-ALL and AML), suggesting B-cell specific functions [75]. SNORA25 is a well-conserved snoRNA and guides the pseudouridylation of residue U801 of the human 18S rRNA subunit (18S-801) [76]. Computational sequence analysis suggested 18S:U814 as another potential target of SNORA25 [39]. However, Deryusheva et al. found that the antisense interaction of SNORA25 to 18S:U814 did not form a functional pseudouridylation pocket within SNORA25 and is therefore unable to modify 18S:U814 [76]. SNORA61 guides the pseudouridylation of the 28S rRNA subunit at residue U2496 [39]. The functional consequences of these rRNA modifications remains to be elucidated.

## 10. Dysregulation of snoRNAs in B-Cell Precursor Acute Lymphoblastic Leukemia (BCP-ALL)

In BCP-ALL, expression of snoRNAs was investigated with Sequencing by Oligonucleotide Ligation and Detection (SOLiD)-based next generation sequencing (NGS) by Teittinen et al. [77]. In total, 46 snoRNAs were differentially expressed between BCP-ALL and T-ALL. Of these snoRNAs, 29 were upregulated and 17 were downregulated. A subset of snoRNAs were validated by qPCR and only scaRNA9 (up) and SNORD24 (down) were significantly differentially expressed in BCP-ALL compared to T-ALL. ScaRNA9, a C/D box scaRNA, guides the 2′-O-methylation of the U2:G19 and U2:A30 on the U2 snRNA [38]. Besides the canonical function, scaRNA9 can be cleaved by DROSHA into an sdRNA with unknown functions. This processing can be enhanced upon stress signaling, such as cisplatin treatment [78]. Unfortunately, differential snoRNA expression between BCP-ALL and normal precursor B-cells was not investigated in this study.

In BCP-ALL, intragenic deletions of the erythroblast transformation-specific related gene (*ERG*) occur in 3–5% of patients [79]. These intragenic deletions occur almost exclusively in BCP-ALL without a recurrent genetic aberration, known as BCP-ALL not otherwise specified (NOS) [80]. This ERG-related subtype is characterized by aberrant expression of DUX4 and ERG transcription factors, which are involved in the differentiation of B-cells [81]. SnoRNA expression analysis showed that SNORD109A, SNORD64, SNORD107 and 12 snoRNAs in the SNORD116 cluster (SNORD116-11, 14–18, 20–24, 27) were upregulated in ERG-related BCP-ALL patients compared to non-ERG-related BCP-ALL NOS patients [80]. Most upregulated SNORD116 family members belong to the snoRNA group (SNOG)-2 of SNORD116 snoRNAs. All these snoRNAs are genomically located in the 15q11-q13 region [82], which deletion is associated with various myelocytic malignancies, such as AML and Chronic Myelomonocytic Leukemia (CMML) [83]. These findings may suggest a role for the 15q11-13 region in lineage specific differentiation. Furthermore, the gene coding the multifunctional protein necdin (*NDN*) is located in the 15q11-13 region and NDN plays an important role during hematopoietic regeneration by inhibiting excessive HSC proliferation [84]. This was confirmed in a *NDN*-deficient mice model, which exhibited an enhanced number of proliferating HSCs [84].

In addition to the profiling of snoRNAs, the expression of critical compounds of the rRNA methylation complex, including FBL, NOP56, NOP58, NHP2L1, nucleolin (NCL), and cMYC, which is a regulator of C/D box snoRNA expression, was studied in pediatric BCP-ALL [85]. In this study, no significant correlations between the expression of the compounds of the rRNA methylation complex and leukemic blast counts in peripheral blood were found. Interestingly, expression levels of FBL, NOP56, cMYC, SNORD35b, and SNORD46 were significantly higher in patients who experienced relapse [85]. In addition, high expression of FBL, cMYC, SNORD35B, and SNORD46 was found to be associated with reduced leukemia free survival, indicating that these factors may be used as prognostic markers. SNORD46 is upregulated in multiple types of cancer [86], whereas inhibition of SNORD46 in A549 cells led to a decrease in cell viability, migration and tissue invasion, suggesting an oncogenic role for SNORD46 in pediatric BCP-ALL.

## 11. Dysregulation of snoRNAs in CLL

CLL is the most common adult leukemia in the Western world [87]. It is characterized by the clonal expansion of CD5+ B-cells and mainly elderly people are affected [88]. CLL is a heterogeneous disease and most patients survive decades, while others have a rapid disease progression with poor outcome [89]. In a study of Ronchetti et al., snoRNA expression was profiled in CLL patients and different snoRNAs were identified as potential biomarkers [74]. SNORD116-1, SNORD116-23, SNORD116-29, SNORD94, and SNORA36A were expressed at low levels in CLL and N, MZ and SM B-cells compared to GC B-cells, suggesting that CLL cells are not from GC origin. SNORA6, SNORA31, SNORA62, SNORA71C, SNORD37, and SNORD50B were significantly downregulated in CLL compared to normal total tonsillar B-cells. Downregulation of SNORD50B has also been observed in a variety of other human cancers, including breast cancer [90], lung cancer [91], melanoma [91] and T-cell lymphoma [91]. The expression of SNORA31 in CLL patients correlated with the expression of its host gene, tumor protein translationally controlled 1 (TPT1), which is an important target of TP53 [92]. Thus, reduced TP53 activity in CLL may explain the low SNORA3 expression. SNORD50A and SNORD50B both directly bind and inhibit *K-RAS*. Deletion of these snoRNAs leads to an increased activity of oncogenic K-RAS signaling, suggesting a functional role for loss of these snoRNAs in CLL development [90,91]. In addition, the snoRNA host gene 5 (SNHG5), which harbors SNORD50A/B, is confirmed to regulate chemotherapy resistance by modulating the SNHG5/miR-32/DNAJB9 axis in AML patients [93]. Also in CML, SNHG5 is associated with chemotherapy resistance by modulating miR-205-5p function [92]. Whether SNHG5 also plays a role in CLL remains to be analyzed.

Ronchetti et al. also compared the expression of snoRNAs in different genetic subgroups of CLL. They showed that CLL patients with a deletion of chromosome 11q23 (del11) have reduced expression of scaRNA9 compared to other CLL subgroups, which can be explained by its location within the deleted region [74]. In addition, SNORA70F was downregulated by unknown mechanisms in CLL patients with trisomy 12 (12+), del11, and CLL patients which are ZAP-70 positive or CD38 positive [74]. In immunoglobulin heavy chain gene (IGHV) unmutated CLL (UM-CLL), the expression of SNORA70F and SNORA70C was decreased, whereas SNORA71C levels were increased compared to IGHV mutated CLL. In addition, CLL with 12+ showed downregulation of scaRNA17 and upregulation of SNORA2b, SNORD59a and SNORD59b compared to non-12+ CLL patients, which can be explained by the extra chromosome 12. Finally, CLL patients with deletion of chromosome 13p13 (del13) were characterized by 19 downregulated snoRNAs. Strikingly, none of these snoRNAs was located at chromosome 13 [74]. In CLL patients, high expression of SNORA74A and SNORD116-18 and low expression of SNORD56 were significantly associated with shorter progression free survival (PFS) [74], suggesting for a role of these snoRNAs in disease progression. In gastric cancer, knockdown of SNORA74A inhibits cell proliferation [94]. Furthermore, SNORA74B, which shares the same target sites as SNORA74A, contributes to the overactivation of AKT/mTOR signaling, a pathway that is involved in cell survival, proliferation and cell migration [95] and that is frequently aberrant in human cancer [96,97]. These results suggest that overexpression of SNORA74A may increase proliferation and has a potential oncogenic role in CLL.

In a study by Berquet et al. [98], differential expression of SNORD35b, SNORD71, SNORD116-11 and SNORD116-25 was found in CLL compared to normal CD19-positive B-cells. In this study, the majority of the included patients had an adverse prognosis. The difference in patient characteristics and the different cell populations used for the comparative analyses may explain the discrepancies between the differentially expressed snoRNAs determined in this study and the ones found in study of Ronchetti, et al. [74]. In the study of Berquet et al., differentially expressed snoRNAs were also assessed in CLL patients with different chromosomal alterations or mutations in the IGHV. In IGHV-mutated CLL patients two groups with significant different treatment free survival (TFS) (32 versus 144 months) could be identified based on differential expression of 20 snoRNAs. Of these 20 snoRNAs, eight snoRNAs (SNORA12, SNORA22, SNORA27, SNORA56, SNORA70, SNORD8, SNORD105B, and scaRNA8) were upregulated and two snoRNAs (SNORA80 and SNORD1A) were downregulated in both proliferating normal B-cells and proliferating CLL cells, suggesting that these snoRNAs could be functionally relevant for proliferation [98].

Together, these data suggest that snoRNAs are aberrantly expressed in CLL compared to healthy mature B-cells. Furthermore, different cytogenetic aberrations are associated with distinct snoRNA expression profiles. Finally, functional studies indicate a role for snoRNAs in proliferation of CLL cells.

## 12. Dysregulation of snoRNAs in B-Cell Lymphomas

B-cell lymphomas are classified as a variety of hematological diseases originating from mature B-cells [99]. B-cell lymphomas are characterized by chromosomal translocations of regions with important oncogenes and tumor suppressor genes, including C-MYC, Cyclin D1 (CCND1), B-cell lymphoma 2 (BCL2) and B-cell lymphoma 6 (BCL6) [99,100]. In some cases, translocations involve genes that harbor snoRNAs. For instance, a diffuse large B-cell lymphoma patient was identified with t(1;3)(q25;q27), fusing growth arrest-specific transcript-5 (GAS-5) to BCL6, generating the GAS5-BCL6 fusion gene [101]. GAS5 is a noncoding gene that harbors multiple snoRNAs, including SNORD81, SNORD47, SNORD80, SNORD79, SNORD78, SNORD44, SNORD77, SNORD76, SNORD75 and SNORD74 [102]. Despite oncogenic functions of dysregulated GAS5 sno-RNAs in different types of cancer [103,104,105], it remains unknown how t(1;3)(q25;q27) affects the expression levels of GAS5 snoRNAs. The t(3;6)(q27;q15) is another example of a translocation involving snoRNAs in human B-cell lymphoma and involves BCL6 and U50HG (also known as SNHG5) [106,107]. Again, the effect of the t(3;6)(q27;q15) translocation on SNORD50A/B expression has not been studied yet, which is essential to understand a possible role for these snoRNAs in oncogenesis of B-cell lymphoma.

## 13. Dysregulation of snoRNAs in Multiple Myeloma

Multiple myeloma (MM) is an incurable malignancy of plasma cells [108,109] with recurrent chromosomal aberrations in the majority of patients [110]. For instance, the t(4;14)(p16.3;q32.3) is detected in 20% of MM patients and it is associated with reduced overall survival [111,112,113]. This translocation results into the fusion of immunoglobulin heavy chain region enhancer elements to the Wolf–Hirschhorn syndrome candidate 1 gene (WHSC1). WHSC1 encodes 3 protein isoforms, of which two are methyltransferases with H4K20, H3K36, and H3K27 as targets [111]. The t(4;14)(p16.3;q32.3) causes upregulation of WHSC1, but this event is not sufficient to drive oncogenic transformation of plasma cells [111]. ScaRNA22 (ACA11), which is located within the WHSC1 gene, is also upregulated in t(4;14) positive MM. The overexpression of scaRNA22 in t(4;14) positive MM cells was associated with the downregulation of 60S ribosomal protein L13a (RPL13A) and snoRNA U30 [111]. Furthermore, lentiviral overexpression of scaRNA22 resulted in lower levels of H_2_O_2_ in t(4;14)-negative dexamethasone sensitive MM cells (MM1.S) and enhanced MM cell proliferation compared to nontransduced MM1.S cells [111]. The response to oxidative stress (H_2_O_2_) is mediated by the nuclear factor (erythroid-derived 2)-like 2 (NRF2) transcription factor [114]. In normal conditions, NRF2 is located in the cytosol, while during cellular stress conditions with increased levels of reactive oxygen species (ROS), NRF2 translocates to the nucleus where antioxidant genes are transcribed. Mahajan et al. found that mRNA and protein levels of NRF2 were not upregulated after lentiviral scaRNA22 overexpression. However, it was observed that the translocation rate of NRF2 to the nucleus was increased [114]. This could explain the lower ROS levels in scaRNA22 overexpressing t(4;14) MM cells. In addition, scaRNA22 controls ribosome biogenesis in a ROS dependent manner [115] and patients with scaRNA22 overexpression respond better to Bortezomib treatment, suggesting that elevated expression of scaRNA22 is a potential prognostic marker for MM.

In addition to the expression based on cytogenetic subgroups, snoRNA expression was studied at different stages of disease progression, such as monoclonal gammopathy of undetermined significance (MGUS), smoldering MM (SMM), and MM [116,117]. In MGUS, SMM and MM patients, a total of 17 snoRNAs (of which 15 reside in the SNORD115 cluster: SNORD115-3, -4, -5, -6, -7, -9, -10, -11, -12, -13, -23, -24, -25, -32, -44, SNORA64, and SNORA46) were downregulated compared to normal plasma cells [116]. In addition, the expression of eight snoRNAs (SNORA73A, SNORD32A, SNORA68, SNORA5A, SNORD15B, SNORD47, SNORA3, and SNORA71A) were more strongly expressed in patients with more severe disease (MGUS < SMM < MM) [116]. Some of these snoRNAs were functionally investigated. For instance, SNORD32A controlled the response to oxidative stress (H_2_O_2_) and endoplasmic reticulum (ER) stress [118]. An additional study showed that overexpression several snoRNAs was correlated with shorter time to progression in MM patients (SNORD25, SNORD27, SNORD30: median PFS 24 months versus not reached; SNORD31: median PFS 17 months versus 36 months) [117]. These studies indicate that different snoRNAs are dysregulated during disease progression and prognosis of MM and that the prognosis can be predicted based on SNORD25, SNORD27, SNORD30, and SNORD31 expression levels.

## 14. Conclusions and Future Prospective

**Figure 3:**
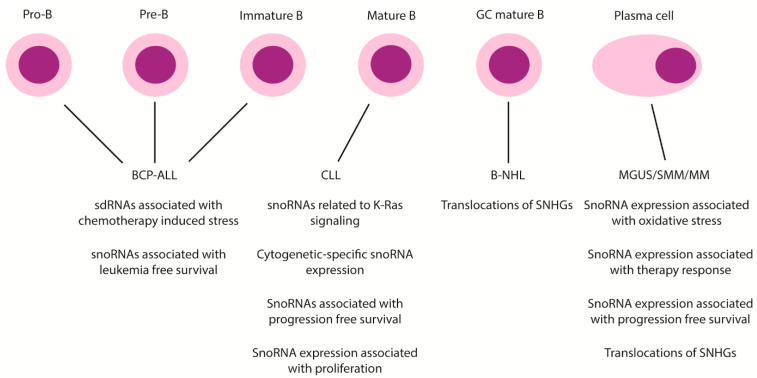
Involvement of snoRNA dysregulation in B-cell malignancies. Types of snoRNA dysregulation per type of B-cell malignancy are shown. Abbreviations: BCP-ALL: B-cell precursor acute lymphoblastic leukemia; CLL: chronic lymphocytic leukemia; B-NHL: B-non-Hodgkin’s lymphoma; MGUS: monoclonal gammopathy of undetermined significance; SMM: smoldering MM; MM: multiple myeloma; SNHG: snoRNA host gene.

As reported above and summarized in Figure 3 and Table 1, several studies have shown that snoRNAs are differentially expressed in different types of B-cell malignancies compared to healthy B-cells or other hematological malignancies. However, most studies consisted of expression profiling analyses without functional characterization of the dysregulated snoRNAs. In addition, the effects of snoRNA dysregulation on the target modifications and additional downstream mechanisms is generally not studied. Furthermore, the functional role of sdRNAs in B-cell malignancies is not elucidated yet.

In different B-cell malignancies, specific snoRNAs were dysregulated. However, the regulation of snoRNA processing and stability is not understood yet. For example, the expression of different snoRNAs of the SNORD116 cluster was dysregulated in ALL and CLL, suggesting the presence of additional, yet unknown mechanisms of snoRNA expression regulation.

Further studies are warranted to enhance our understanding on snoRNAs in B-cell malignancies and to characterize their functions in normal and malignant B-cell differentiation. To achieve this, cellular models with modulated snoRNA expression need to be generated. Overexpression models of snoRNAs can be generated using lentiviral transduction [111]. In addition, novel technologies as CRISPR-Cas9 genome editing allow to generate snoRNA knockout cell and animal models [119]. Furthermore, assays to evaluate modifications of putative snoRNA targets have been developed. 2′O-methylation can be assessed using reverse transcription at low deoxy-ribonucleoside triphosphate (dNTP) concentrations followed by polymerase chain reaction (PCR) (RTL-P) [120]. Pseudouridylation of target RNAs can be studied by treatment with N-Cyclohexyl-N’(2-morpholinoethyl)carbodiimide (CMC) and subsequent reverse transcriptase PCR amplification [121]. In our opinion, these technologies help to reveal the role of snoRNA dysregulation in B-cell malignancies.

## Figures and Tables

**Table 1:** Dysregulated snoRNAs in B-cell malignancies.

Disease	snoRNA	Expression ^a^	Comparison ^a^	Refs.
BCP-ALL	scaRNA9	Up	BCP-ALL vs. T-ALL	[77]
BCP-ALL	SNORD24	Down	BCP-ALL vs. T-ALL	[77]
BCP-ALL	SNORD109A, SNORD64, SNORD107	Up	ERG-related BCP-ALL vs. non-ERG related BCP-ALL	[80]
BCP-ALL	SNORD116-11, 14–18, 20–24, 27	Up	ERG-related BCP-ALL vs. non-ERG related BCP-ALL	[80]
BCP-ALL	SNORD35B, SNORD46	Up	Relapse vs. complete remission	[85]
CLL	SNORA6, SNORA31, SNORA62, SNORA71C, SNORD37, SNORD50B	Down	CLL vs. total tonsillar B-cells	[74]
CLL	scaRNA9	Down	CLL with (del11) vs. other subtypes	[74]
CLL	SNORA70F	Down	CLL with (12+), (del11), or ZAP70+ and CD38+ vs. others	[74]
CLL	SNORA70F, SNORA70C	Down	UM-CLL vs. M-CLL	[74]
CLL	scaRNA17	Down	CLL with (12+) vs. CLL non-(12+)	[74]
CLL	SNORA2B, SNORD59A, SNORD59B	Up	CLL with (12+) vs. CLL non-(12+)	[74]
CLL	SNORA74A, SNORD116-18	Up	High expression associated with shorter PFS	[74]
CLL	SNORD56	Down	Low expression associated with shorter PFS	[74]
CLL	SNORD116-11, -25	Up	CLL vs. CD19+ cells	[98]
CLL	SNORD35B, SNORD71	Down	CLL vs. CD19+ cells	[98]
MM	scaRNA22	Up	t(4;14) positive MM vs. t(4;14) negative MM	[111]
MM	SNORD115-7, -23, -5, -44, -25, -6, -24, -4, -3, -9, -11, -32, -10, -12, -13, SNORA46, SNORA64	Down	MGUS, SMM and MM vs. normal plasma cells	[116]
MM	SNORA73A, SNORD32A, SNORA68, SNORA5A, SNORD15B, SNORD47, SNORA3 AND SNORA71S	Up	Higher expression during disease progression (MGUS < SMM < MM)	[116]

^a^ Expression up- or downregulated in the first group as compared to the second group; Dysregulated snoRNAs in different types of B-cell leukemia are listed. Abbreviations: BCP-ALL: B-cell precursor acute lymphoblastic leukemia; CLL: chronic lymphocytic leukemia; B-NHL: B-non-Hodgkin’s lymphoma; MGUS: monoclonal gammopathy of undetermined significance; SMM: smoldering MM; MM: multiple myeloma; SNHG: snoRNA host gene.

## Data Availability

Not applicable.
